# An ERP study of effects of regularity and consistency in delayed naming and lexicality judgment in a logographic writing system

**DOI:** 10.3389/fpsyg.2014.00315

**Published:** 2014-04-14

**Authors:** Yen Na Yum, Sam-Po Law, I-Fan Su, Kai-Yan Dustin Lau, Kwan Nok Mo

**Affiliations:** ^1^Division of Speech and Hearing Sciences, University of Hong KongHong Kong, China; ^2^Department of Chinese and Bilingual Studies, The Hong Kong Polytechnic UniversityHong Kong, China

**Keywords:** phonological regularity, phonological consistency, Chinese, delayed naming, lexical decision, event-related potential (ERP)

## Abstract

Phonological access is an important component in theories and models of word reading. However, phonological regularity and consistency effects are not clearly separable in alphabetic writing systems. We investigated these effects in Chinese, where the two variables are operationally distinct. In this orthographic system, regularity is defined as the congruence between the pronunciation of a complex character (or phonogram), and that of its phonetic radical, while phonological consistency indexes the proportion of orthographic neighbors that share the same pronunciation as the phonogram. In the current investigation, regularity and consistency were contrasted in an event-related potential (ERP) study using a lexical decision (LD) task and a delayed naming (DN) task with native Chinese readers. ERP results showed that effects of regularity occurred early after stimulus onset and were long-lasting. Regular characters elicited larger N170, smaller P200, and larger N400 compared to irregular characters. In contrast, significant effects of consistency were only seen at the P200 and consistent characters showed a greater P200 than inconsistent characters. Thus, both the time course and the direction of the effects indicated that regularity and consistency operated under different mechanisms and were distinct constructs. Additionally, both of these phonological effects were only found in the DN task and absent in LD, suggesting that phonological access was non-obligatory for LD. The study demonstrated cross-language variability in how phonological information was accessed from print and how task demands could influence this process.

## Introduction

All writing systems carry phonological information, but they vary in the nature of correspondence between orthographic units, e.g., whole word, sublexical components, and phonological units, e.g., phonemes, rimes, syllables. For instance, in alphabetic scripts such as English, French, German, and Korean *hangul*, the orthography-phonology mapping is between letters and phonemes; the correspondence in systems such as Japanese *katakana* and *hiragana* is between a symbol (i.e., kana) and a syllable (or more precisely mora); and in the case of Chinese, each character, considered a logograph by some, is associated with a syllable. These cross-linguistic variations are expected to have profound impact on how phonological information is accessed from print and implications for models of reading.

Given the existence of sublexical correspondence, all theoretical models of reading in alphabetic scripts assume a non-lexical reading mechanism without necessarily a lexical route (e.g., Coltheart, 1978; Hillis and Caramazza, 1995; Plaut et al., 1996). The orthography-phonology relationships can be characterized in terms of regularity and consistency, depending on the theoretical approach. The regularity of a word is determined by whether its pronunciation conforms to grapheme-phoneme correspondence (GPC) rules of the language (e.g., regular words such as *raid*, *pink* vs. irregular words such as *pint*, *have*; Coltheart et al., 1993, 2001), while the consistency of a word depends on the strength of spelling-sound connections derived from the properties of the pronunciations of the “body” of other similarly spelled words (e.g., consistent words such as *bust*, *dust*, *gust*, *just*, *lust*, *must*, *rust* vs. inconsistent words such as *cost*, *host*, *lost*, *most*, *post*; Seidenberg and McClelland, 1989; Plaut et al., 1996). Both regularity and consistency have been shown to affect naming latency. Irregular words take longer to name than regular words (e.g., Baron and Strawson, 1976; Gough and Cosky, 1977; Stanovich and Bauer, 1978), and the effect is more pronounced in low frequency words (e.g., Andrews, 1982; Seidenberg et al., 1984; Waters et al., 1984). Readers are also slower to read aloud inconsistent than consistent lexical items (Glushko, 1979). However, regularity and consistency are not easily distinguishable. Irregular or exception words are often inconsistent; moreover, in some studies regularity is defined in terms of neighborhood characteristics such as the relative numbers of friends (e.g., peak-teak) and enemies (e.g., peak-pear) (e.g., Peereman, 1995). In the few studies that have manipulated both regularity and consistency, effects of consistency are robust while those of regularity are unclear or limited (Andrews, 1982; Kay and Bishop, 1987; Cortese and Simpson, 2000; Jared, 2002). This has raised the question whether regularity effects conceptualized as GPC knowledge have important impact on reading alphabetic scripts. Interestingly, although the Chinese writing system is generally considered logographic, the notions of regularity and consistency have been shown to be highly relevant to reading, and they are theoretically more distinct by comparison. Hence, the present study investigated their underlying mechanism using a technique known for its excellent temporal resolution and ability to reveal online unfolding of cognitive processes, i.e., event-related potential (ERP), in addition to the traditional behavioral measures.

Almost all Chinese characters are monosyllabic and correspond to morphemes. As such, the Chinese script is described as a morphosyllabic system. Given that there are no elements within a character that correspond to phonemes or tone, the postulation of a non-lexical reading pathway in Chinese may be irrelevant. Nonetheless, more than 80% of all Chinese characters are phonograms consisting of components that carry some semantic and phonological information of the character. Orthographically, Chinese characters are made up of spatial arrangements of strokes, which combine to form larger units called “radicals.” Radicals may further combine to form complex characters or phonograms. Phonograms contain a semantic radical and a phonetic radical providing a clue to the meaning of a character and one to the pronunciation of the character, respectively. For instance, the character 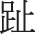 *zi2* “toe” has a semantic radical 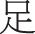 on the left meaning “foot” and a phonetic radical 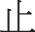 *zi2* on the right. (In this paper, phonetic transcriptions of Chinese characters are given in *jyutping*, a romanization system developed by the Linguistics Society of Hong Kong. The number in the transcription represents the tone.) According to the entries in two dictionaries of Cantonese phonograms (Ni, 1982; Li, 1989), Law et al. (2009) reported that about 34–40% of phonograms are “regular” characters. Their pronunciations are segmentally identical (regardless of tone) to the pronunciation of their phonetic radical when it occurs as a character (e.g., 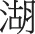 *wu4* and 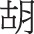 *wu4*). Another 30% are “partially regular” phonograms sharing at least the same rime as their phonetic radical (e.g., 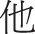 *taa1* and 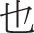 *jaa5*), and the rest are “irregular” with no phonological relationship with their phonetic radical (e.g., 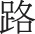 *lou6* and 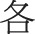 *gok3*). Most phonetic radicals are also existing characters, such as 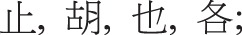; however, it is important to note that there is a non-negligible number of phonetic radicals, approximately 16% of all phonetic radical entries listed in Li (1989), that do not exist alone, e.g., the right-hand components of 
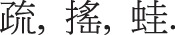
. These radicals have no associated phonological representations or meaning.

Besides regularity, the phonological property of a character can also be described in terms of consistency. It refers to the extent to which the phonetic radical serves as a reliable cue to the pronunciations of the phonograms containing it. A character of high consistency is one that sounds the same as most, if not all phonograms sharing the same phonetic radical (e.g., 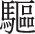 *keoi1*, 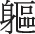 *keoi1*, 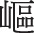 *keoi1*, 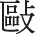 *keoi1*, 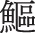 *keoi1*, 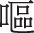 *ngau2*), and a low consistency character is one that shares the same phonetic radical with phonograms that sound differently (e.g., 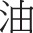 *jau4*, 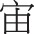, *zau6*, 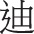 *dik6*, 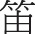 *dek6*, 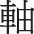 *zuk6*). In other words, regularity is defined by the phonological distance between a phonogram and its phonetic radical and only applicable to phonograms with phonetic radicals that exist as stand alone characters, while consistency is determined by the number of different phonological forms associated with a family or neighborhood of phonograms having a common phonetic radical. One can see that consistency in Chinese is comparable to that in alphabetic scripts (Lee, 2008), whereas regularity has a distinct definition.

Psycholinguistic studies of character recognition has accumulated ample evidence that phonetic radicals access phonological representations independently of and in parallel with the phonograms (e.g., Seidenberg, 1985; Hue, 1992; Wu et al., 1994; Weekes et al., 1998; Zhou and Marslen-Wilson, 1999; Ding et al., 2004). Low frequency regular phonograms, but not high frequency ones, have significantly shorter reading latencies than irregular phonograms. Similarly, in a series of reading aloud experiments, Fang et al. (1986) demonstrated effects of consistency. Regular/consistent characters were named significantly faster than regular/inconsistent and irregular phonograms. Comparable findings were reported in Lian (1985) and more recently in Lee et al. (2005). Lee et al. manipulated character frequency, regularity and consistency. A significant interaction between regularity and consistency was found for low frequency characters; furthermore, consistency effects were observed among irregular but not regular phonograms. Irregular high consistency characters were named faster than irregular low consistency characters. These findings suggest that regularity and consistency are independent variables. It is, however, worth noting that these observations were based on a very small set of stimuli of 10 in each experimental condition.

Besides behavioral evidence, the “ortho-phonological” effects can also be observed in neural responses of specific ERP components. Using the homophone judgment task in which stimuli contained phonetic radicals varying in consistency, Lee and colleagues found effects of consistency in N170 in the temporo-occipital region, P200 in the frontal region, and N400 in the central region. In particular, greater negativity in N170 and greater positivity in P200 were elicited by inconsistent characters, compared with consistent ones, while greater N400 was found for consistent than inconsistent characters (Lee et al., 2007). The effects at N170 and P200 were interpreted as early extraction of phonological information from the phonetic radical, whereas effects at N400 were taken to reveal post-lexical processing resulting from competition among activated representations at the lexical level. Somewhat different results were reported when pseudo-characters were employed. Although pseudo-characters containing unpredictable (inconsistent) phonetic radicals exhibited greater P200, they also elicited greater N400 (Lee et al., 2006a). When consistency was manipulated with neighborhood characteristics taken into consideration, including orthographic neighborhood (number of phonograms sharing the same phonetic radical), phonological alternatives (number of different phonological forms associated with a phonetic radical family), and phonological neighborhood (number of homophonic characters associated with a phonological form), a more fine-grained picture emerged (Hsu et al., 2009). High consistency characters exhibited greater negativity in N170, smaller P200, and greater N400 compared with low consistency stimuli, but these effects were restricted to characters from large orthographic neighborhoods (*N* > 10) compared with small ones (*N* < 4).

It is notable that the aforementioned studies focused on the consistency effect. The only ERP study that has involved regularity was Lee et al. (2006b). In a character recognition task, participants were presented with pairs of prime-target characters varying in stimulus-onset-asynchrony (SOA) and semantic relatedness. Among the three conditions in which the prime and target are semantically unrelated, two of them involved a phonogram prime containing a phonetic radical that is semantically related to the target but these two conditions differed in terms of whether the prime was a regular or irregular phonogram (e.g., Regular phonogram: 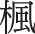 (prime) *fung1* “maple” (phonetic radical 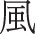 *fung1* “wind”) → 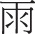 (target) *jyu5* “rain”; Irregular phonogram: 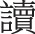 *duk6* “read” 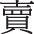 *maai6* ‘sell’) → 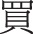 *maai5* “buy”). Both conditions revealed significant N400 semantic priming effects when contrasted with the unrelated control condition but only in the shorter SOA conditions (50 and 100 ms). Moreover, the N400 effect elicited by the regular phonograms appeared earlier and persisted longer than the irregular phonograms. These results suggest that the phonological forms of the phonogram and its phonetic radical have modulating effects on semantic processing during the N400 time window.

In summary, few ERP studies have examined regularity and consistency simultaneously and how they may differ in neural representation. Moreover, the contrast in consistency in previous work was often between extreme values, especially for high consistency characters with an average consistency approaching 1. Little information was provided on the composition of the high and low consistency characters with respect to their regularity status. In other words, it is not clear whether stimuli in the two consistency conditions had comparable number of regular and irregular characters. Hence, consistency might have been confounded with regularity in previous ERP works.

Given the conceptual distinction between “word-based” regularity and “neighborhood-based” consistency, it is reasonable to expect that they differ in neural correlates, at least in terms of time course. To illustrate, upon seeing a medium-to-high frequency phonogram containing a free-standing phonetic radical, a skilled reader may be able to immediately segment the character into radical components and access the corresponding phonological forms, i.e., the whole character and the phonetic radical. The phonetic radical then spreads activation to phonograms containing it; the activated phonograms then access their phonological representations, which compete with one another. Such a scenario is compatible with most models of reading in Chinese (e.g., Taft and Zhu, 1997; Perfetti et al., 2005). It also predicts that the regularity effect may emerge earlier *and* last longer than the consistency effect. The former effect results from competition between two phonological forms activated by direct orthography-to-phonology mapping, while the latter arises from competition among phonograms activated by the segmented phonetic radical. Moreover, we hypothesize that the consistency effect has a shorter time course than regularity. Competition between a phonogram and its phonetic radical is driven by orthographic forms of the stimulus, and therefore, persists until a selection for output is made. In contrast, phonograms in an orthographic neighborhood are activated “indirectly” by the phonetic radical in the stimulus, and the majority of the activated representations do not correspond to the target. These predictions differ importantly from previous findings by Lee and colleagues, which would predict consistency effects in the time windows of N170, P200, and N400, and regularity effects occurring mainly in N400.

The current study employed behavioral and neural measures of regularity and consistency. Given the impact of the characteristics of orthographic and phonological neighborhoods on character naming, and the difficulties in identifying enough lexical items varying in regularity and consistency while matched on neighborhood variables, effects of regularity and consistency were studied separately using different sets of stimuli. In addition to using a task that explicitly accesses phonological information, i.e., reading aloud characters but after a delay to eliminate movement artifacts undesirable in ERP experiments, a lexical decision (LD) task was administered. Lexicality judgment is probably the most common task in lexical processing research. While not central to our research questions, performance in lexicality judgment ensures that participants attend to the stimuli and the experimental task. Previous studies have shown enhanced N400 to pseudowords compared to real words, interpreted as reflecting difficulty in lexical access (Bentin et al., 1985; Holcomb, 1993; Nobre and McCarthy, 1994). Although lexicality can be determined without recourse to phonology, the presence or absence of phonological effects in such as task has both theoretical and practical significance. Most theoretical models assume that access to phonology is automatic upon seeing a written word without reference to the goal(s) of a task. A comparison between reading aloud and LD will allow us to see if the reading processes involved change as a function of task demands. If phonological information is available automatically in lexicality judgment and the effects are of comparable strength to naming, then LD would be preferred especially in reading experiments using ERPs, because responses are based on single stimuli as opposed to pairs of stimuli in homophone judgment and relatively free of motion artifacts.

## Materials and methods

### Participants

Twenty four (12 females) right-handed native Cantonese speakers aged 18–26 (*M* = 21.17, *SD* = 1.97) with normal neurological profile and visual acuity were recruited for this study. Participants received cash compensation upon completion of experimental tasks. Written informed consent was obtained from all participants and the experiments were approved by the Human Research Ethics Committee for Non-Clinical Faculties of the University of Hong Kong.

### Materials

Real word stimuli consisted of 160 phonograms written in traditional Chinese script of left-right or top-bottom configuration with one phonetic and one semantic radical. In the LD task, 160 pseudo-characters were used as well. These were created by randomly combining the phonetic and semantic radicals of the real character stimuli in accordance to orthographic rules. Pseudo-characters and real characters were matched on structural configuration and stroke number.

Two lists of characters selected from the real words were used to investigate the effects of phonological regularity and consistency in LD and Delayed Naming (DN) tasks. Regularity was defined by the relationship between the pronunciation of the character and that of its phonetic radical. Regular characters shared both onset and rime with its phonetic radical regardless of tone, while irregular characters did not meet this criterion. One common method to calculate a consistency value is to divide the number of friends (orthographic neighbors that share the same pronunciation) by the total number of orthographic neighbors. This proportion is known as type consistency. Another way of measuring consistency, known as token consistency, takes into account the lexical frequency of the orthographic neighbors, i.e., giving more weight to neighbors with higher frequency. Note that the measure of regularity is only meaningful when the phonetic radical is an existing character that carries its own pronunciation, while consistency does not have this limitation. In addition, the phonetic radical was not counted as a neighbor in our calculations of type or token consistency values; this is different from the estimates of consistency in Lee and colleagues' work.

In our stimuli, regular and irregular characters (*n* = 55 in each condition) were matched in stroke number, lexical frequency, orthographic neighborhood size, type and token phonological consistency, number of homophones, number of syllables associated with an orthographic neighborhood, and lexical frequency of the phonetic radical. Consistent and inconsistent characters (*n* = 36 in each condition) were significantly different in type as well as token consistency. They also differed in the number of phonological alternatives for the phonetic radical, as inconsistent characters tended to have more orthographic neighbors with different pronunciations. Importantly, for both consistent and inconsistent items, half were regular and half were irregular. They were also matched in stroke number, lexical frequency, orthographic neighborhood size, number of homophones, and standalone frequency of the phonetic radical. Properties of the stimuli used in each condition are shown in Table 1.

**Table 1:** **Properties of the stimuli in the regularity and the consistency contrasts**.

	**Irregular (*N* = 55)**		**Regular (*N* = 55)**		
	**Range**	**Mean (*SD*)**	**Range**	**Mean (*SD*)**	***p*-value**
Stroke	6–20	11.76 (2.96)	6–20	11.58 (3.34)	0.76
Frequency (per mil.)	0.31–1306.43	333.67 (386.29)	4.23–2075.05	325.87 (419.34)	0.92
Family size	3–15	7.16 (3.18)	3–15	6.96 (3.05)	0.74
Consistency (Type)	0.07–0.75	0.33 (0.20)	0.07–1	0.35 (0.26)	0.61
Consistency (Token)	0.01–0.99	0.5 (0.36)	0.01–1	0.45 (0.36)	0.48
No. of homophones	0–16	4.42 (3.98)	0–22	4.67 (4.82)	0.76
No. of associated syllables	2–12	4.40 (2.20)	1–8	4.40 (1.99)	1
Radical frequency (per mil.)	3.13–5616.44	607.58 (1121.61)	11.98–4090.13	788.91 (944.54)	0.36
	**Consistent (*N* = 36)**		**Inconsistent (*N* = 36)**		
Stroke	7–20	12.44 (3.26)	7–20	12.08 (3.76)	0.66
Frequency (per mil.)	5.92–1299.44	237.79 (331.07)	7.93–1053.11	306.56 (331.53)	0.38
Family size	5–8	6.31 (0.86)	4–9	6.56 (1.38)	0.36
Consistency (Type)	0.13–1	0.53 (0.28)	0.13–0.50	0.21 (0.09)	<0.001
Consistency (Token)	0.09–1	0.71 (0.32)	0.01–0.80	0.22 (0.21)	<0.001
No. of homophones	0–15	4.81 (3.62)	0–19	4.06 (4.93)	0.46
No. of associated syllables	1–4	2.75 (1.05)	4–8	5.22 (0.93)	<0.001
Radical frequency (per mil.)	0.63–4090.13	665.70 (1039.60)	0–1917	408.15 (495.87)	0.19

### Procedure

After informed consent, participants were seated in an electrically and acoustically shielded room. Stimuli were presented on a computer screen located approximately 60 cm away. For all participants, a LD was administered followed by a delayed naming task (DN). A practice block was given to each participant prior to each task. On each trial of LD, a fixation cross (500 ms) preceded a yellow character (100 × 90 pixels) was presented for 800 ms (1200–1500 ms ITI) on a black background. Participants decided if the character was real by pressing a button for real characters and another button for pseudo-characters. The stimuli were given in six blocks in a random sequence delivered by E-Prime (Psychology Software Tools Inc., USA), with the response buttons counterbalanced across participants.

In DN, only real characters were shown. On each trial, a fixation cross was presented for 500 ms and the character was displayed for 800 ms. Then the character would be replaced by three asterisks, which remained on the screen until a response was made. Participants were instructed to name the displayed character upon seeing the asterisks. ERP measurement was time-locked to the visual onset of characters, prior to actual utterance. The response delay served to reduce muscle artifacts produced during verbal production. The responses were recorded and coded offline for response accuracy.

#### EEG recordings

The EEG data were recorded from 64 Ag/AgCl electrodes (10–20 system) with a common vertex reference electrode located between electrodes Cz and CPz, and ground (GND) positioned anterior to electrode Fz. Vertical and horizontal eye movements were monitored by bipolar electrodes (VEOG) placed on the supra- and infraorbital ridges of the left eye and bipolar electrodes (HEOG) placed on the left and right side of the lateral orbital rim. Electrode impedance was maintained below 5 KΩ and data were digitized online at 1 kHz with a band pass filter of 0.05–200 Hz using SynAmps2® (Neuroscan, Inc., El Paso, TX, USA) amplifiers.

#### ERP data processing

In the off-line analysis, continuous data were filtered using a zero phase shift low-pass filter of 30 Hz (12 dB/octave slopes). Channels affected by eye blink artifacts were corrected using a model artifact implemented in Scan 4.5 software (Neuroscan, Inc), with a minimum of 100 eyeblink artifacts for each participant. Segments of −200 to 1000 ms post-stimulus onset intervals were later extracted and baseline corrected using the pre-stimulus intervals (−200 to 0 ms). Trials with incorrect responses, muscle artifacts, or voltage exceeding 100 μ V were automatically rejected. The remaining data were re-referenced to the average of the two mastoid electrodes and used to compute grand average waveforms for each condition.

### Statistical analyses

For behavioral effects of lexicality, *t*-tests were used to compare accuracy and response time (RT) to real and pseudo-characters in LD. For consistency and regularity effects, *t*-tests were performed on the accuracy and RT data in LD. Since a response delay was introduced in DN, only effects on naming accuracy were examined in this task.

In both LD and DN, mean amplitudes of the N170, P200, and N400 ERP components timelocked to character onset were examined and analyzed statistically. Three-Way ANOVAs were conducted for each of the three components, with Electrode Location (N170: P5, P6, P7, P8, PO5, PO6, PO7, PO8; P200: FC3, FC4, C3, C4, CP3, CP4; N400: FC5, FC6,C5, C6, CP5, CP6,P5, P6) and Hemisphere (left vs. right) as within-subject independent variables in addition to Tasks (LD vs. DN) and Experimental Conditions (Consistent vs. Inconsistent or Regular vs. Irregular). We chose these electrode locations based on previous ERP works on these phonological effects in Chinese (Lee et al., 2007; Hsu et al., 2009). Estimation of analysis windows was based on the peak latencies derived from the mean amplitude for all trials at the selected electrode locations. The window for N170 was set as 100–200 ms, with the peak at 151 ms. The P200 window was 200–270 ms, with the peak at 233 ms. The N400 window was 270–400 ms, with the peak at 326 ms. These time windows were roughly comparable to previous findings (Lee et al., 2007; Hsu et al., 2009). The lexicality effect in LD was examined with all real characters and pseudo-characters at the N400 component using the same electrode locations and time window. The significance threshold for *post-hoc* ANOVAs was corrected for multiple comparisons using Bonferroni adjustment.

## Results

### Behavioral results

A summary of the behavioral findings is shown in Table 2. In LD, trials with response latencies below 200 ms and exceeding 2000 ms were discarded (<1%), and incorrect trials were excluded in RT calculations. For lexicality effects in LD, participants responded more accurately to real characters (*M* = 97%, *SD* = 0.02) than to pseudo-characters [*M* = 89%, *SD* = 0.09; *t*_1(23)_ = 3.53, *p* = 0.002; *t*_2(159)_ = 7.12, *p* < 0.001 where *t*_1_ denotes results from subject analyses and *t*_2_ denotes results from item analyses]. Participants were also faster when responding to real characters (*M* = 546 ms, *SD* = 58.50) compared to pseudo-characters [*M* = 661 ms, *SD* = 123.98; *t*_1(23)_ = 6.07, *p* < 0.001; *t*_2(159)_ = 22.94, *p* < 0.001].

**Table 2:** **Behavioral results in lexical decision and delayed naming, standard deviations are given in parentheses**.

	**Regular**	**Irregular**	**Consistent**	**Inconsistent**
RT in LD (ms)	546 (55)	553 (66)	551 (65)	542 (54)
				
Accuracy in LD (%)	97.3 (3.1)	97.0 (2.8)	96.2 (3.0)	98.1 (2.8)
Accuracy in DN (%)	98.2 (2.1)	97.3 (2.1)	99.3 (1.2)	97.9 (2.2)

For effects of regularity and consistency on response latencies in LD, a marginal effect of faster RT to regular characters than irregular characters was found in the subject analysis, but this was not significant in the item analysis [*t*_1(23)_ = 1.74, *p* = 0.096; *t*_2(54)_ = 0.78, *p* = 0.441]. Regularity did not have a significant effect on lexicality judgment accuracy [*t*_1(23)_ = 0.58, *p* = 0.567; *t*_2(54)_ = 0.37, *p* = 0.714]. Participants were marginally slower when responding to consistent characters than to inconsistent characters, again only in the subject analysis [*t*_1(23)_ = 1.82, *p* = 0.081; *t*_2(35)_ = 0.90, *p* = 0.373]. Response accuracy was higher for inconsistent than consistent characters, but this was only significant in the subject analysis [*t*_1(23)_ = 2.33, *p* = 0.029; *t*_2(35)_ = 1.52, *p* = 0.136].

In DN, higher accuracy for regular characters than irregular characters was revealed in the subject analysis, but not the item analysis [*t*_1(23)_ = 2.22, *p* = 0.037; *t*_2(54)_ = 0.828, *p* = 0.411]. Higher naming accuracy for consistent than inconsistent characters was shown in the subject analysis only [*t*_1(23)_ = 2.94, *p* = 0.007; *t*_2(35)_ = 1.12, *p* = 0.270].

In short, effects of lexicality on both response accuracy and latency were significant. In contrast, none of the results of the regularity and consistency contrasts were statistically reliable.

### ERP results

**Figure 1:**
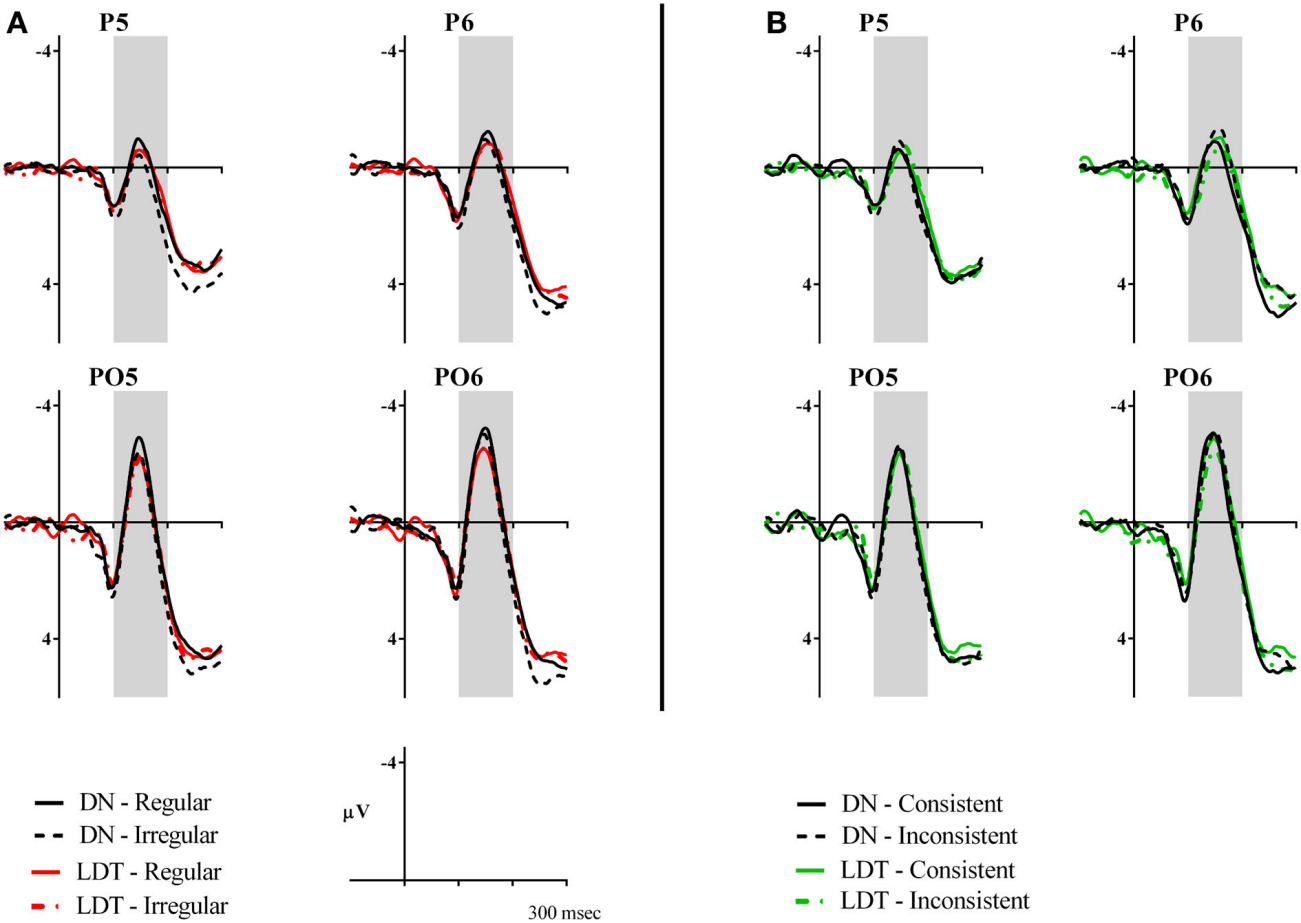
**(A)** Grand average waveforms showing the N170 regularity effect at parietal and parietal-occipital electrodes (P5, P6, PO5, and PO6). Even numbers refer to electrode positions on the right hemisphere, whereas odd numbers refer to those on the left hemisphere. **(B)** Grand average waveforms showing the N170 consistency effect at the same electrodes. Shaded areas represent the analysis window of 100–200 ms.

**Figure 2:**
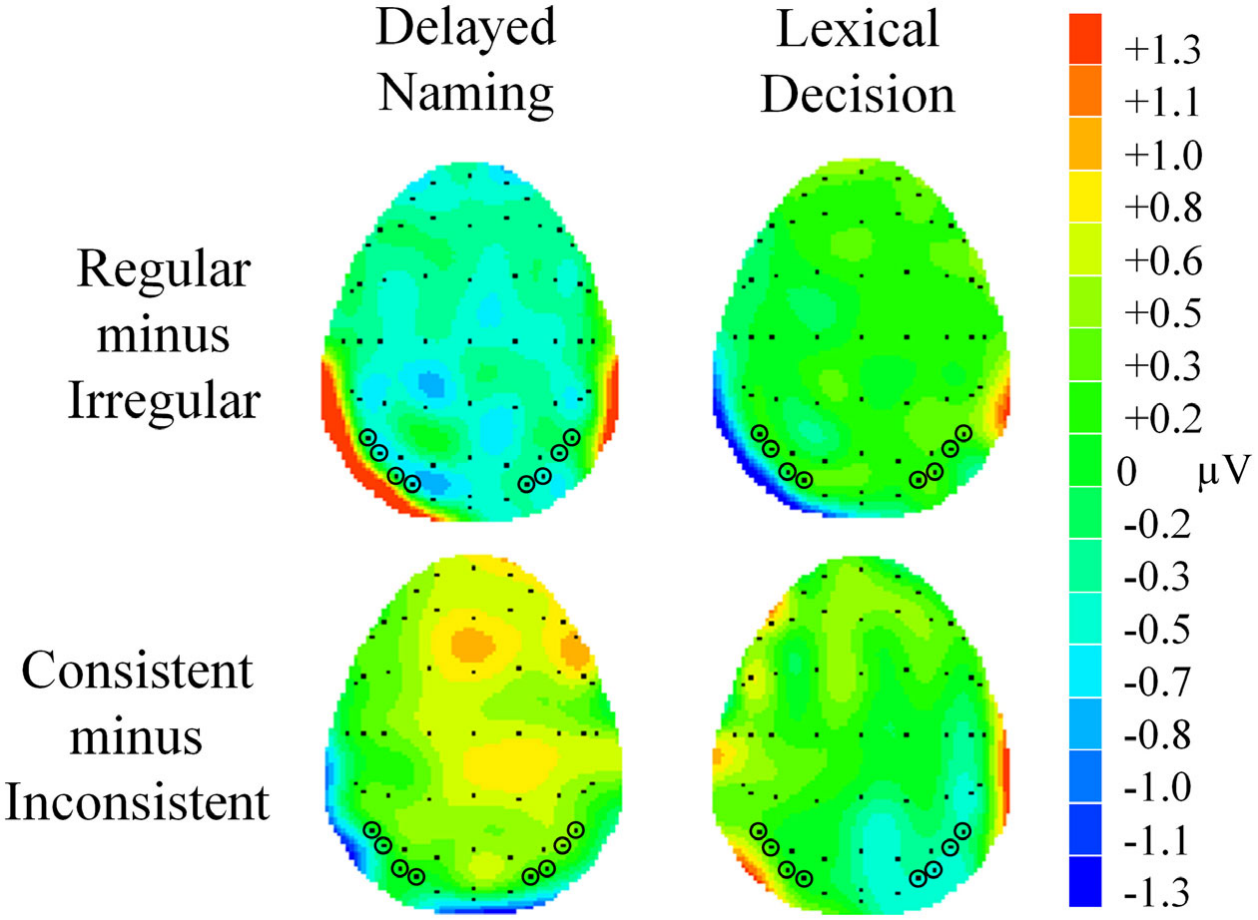
**Voltage maps showing effects of regularity and consistency in delayed naming and lexical decision in N170**. The maps were calculated from the mean difference values (in μV) of regular minus irregular characters and consistent minus inconsistent characters between 100–200 ms. Electrodes P5, P6, P7, P8, PO5, PO6, PO7, and PO8 are circled for reference.

**Figure 3:**
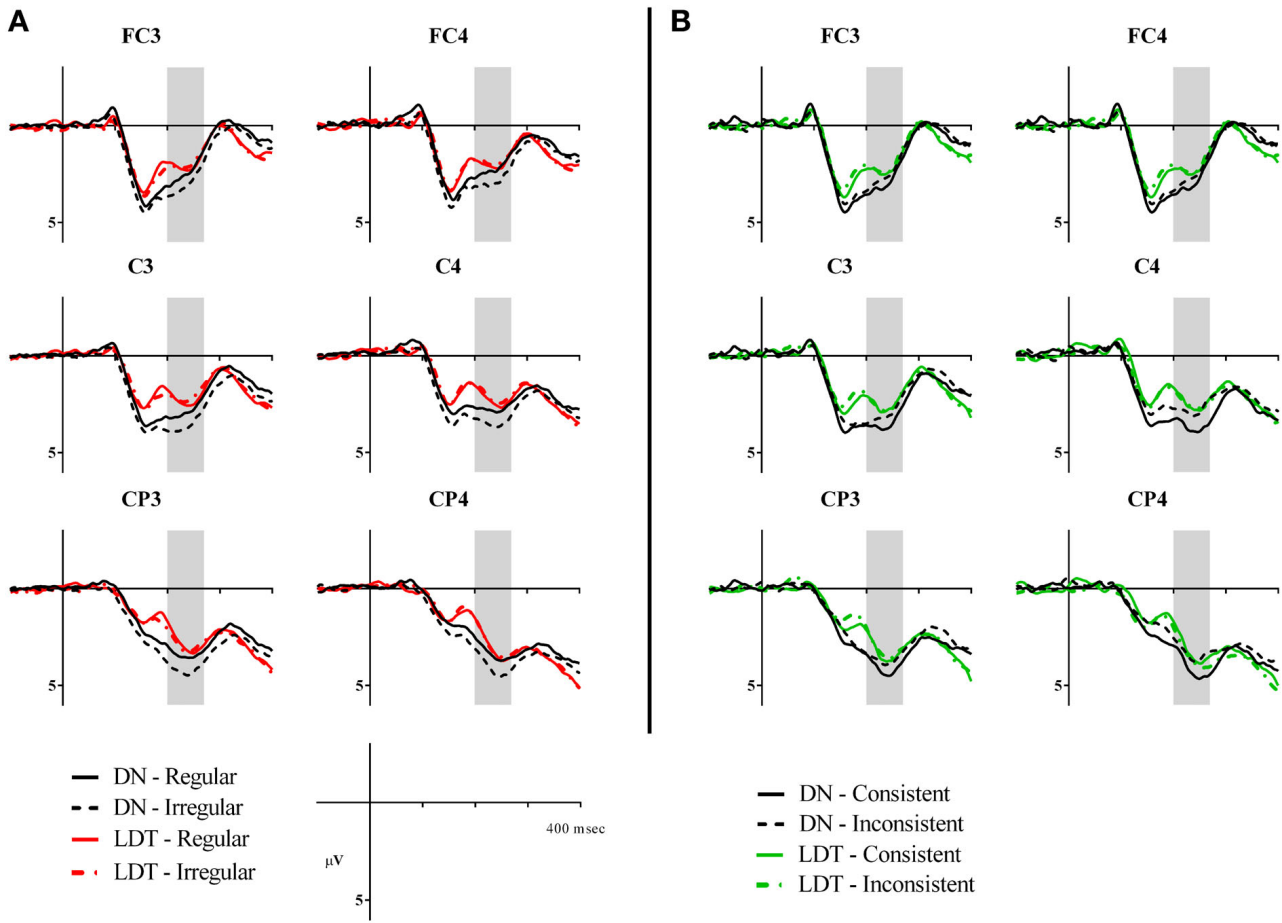
**(A)** Grand average waveforms showing the P200 regularity effect at central sites (FC3, FC4, C3, C4, CP3, and CP4). **(B)** Grand average waveforms showing the P200 consistency effect at the same electrodes. Shaded areas represent the analysis window of 200–270 ms.

**Figure 4:**
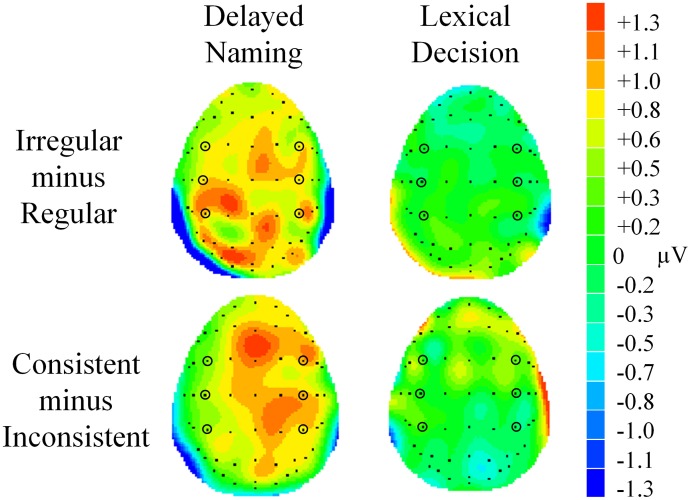
**Voltage maps showing effects of regularity and consistency in delayed naming and lexical decision in P200**. The maps were calculated from the mean difference values (in μV) of irregular minus regular characters and consistent minus inconsistent characters between 200–270 ms. Electrodes FC3, FC4, C3, C4, CP3, and CP4 are circled for reference.

**Figure 5:**
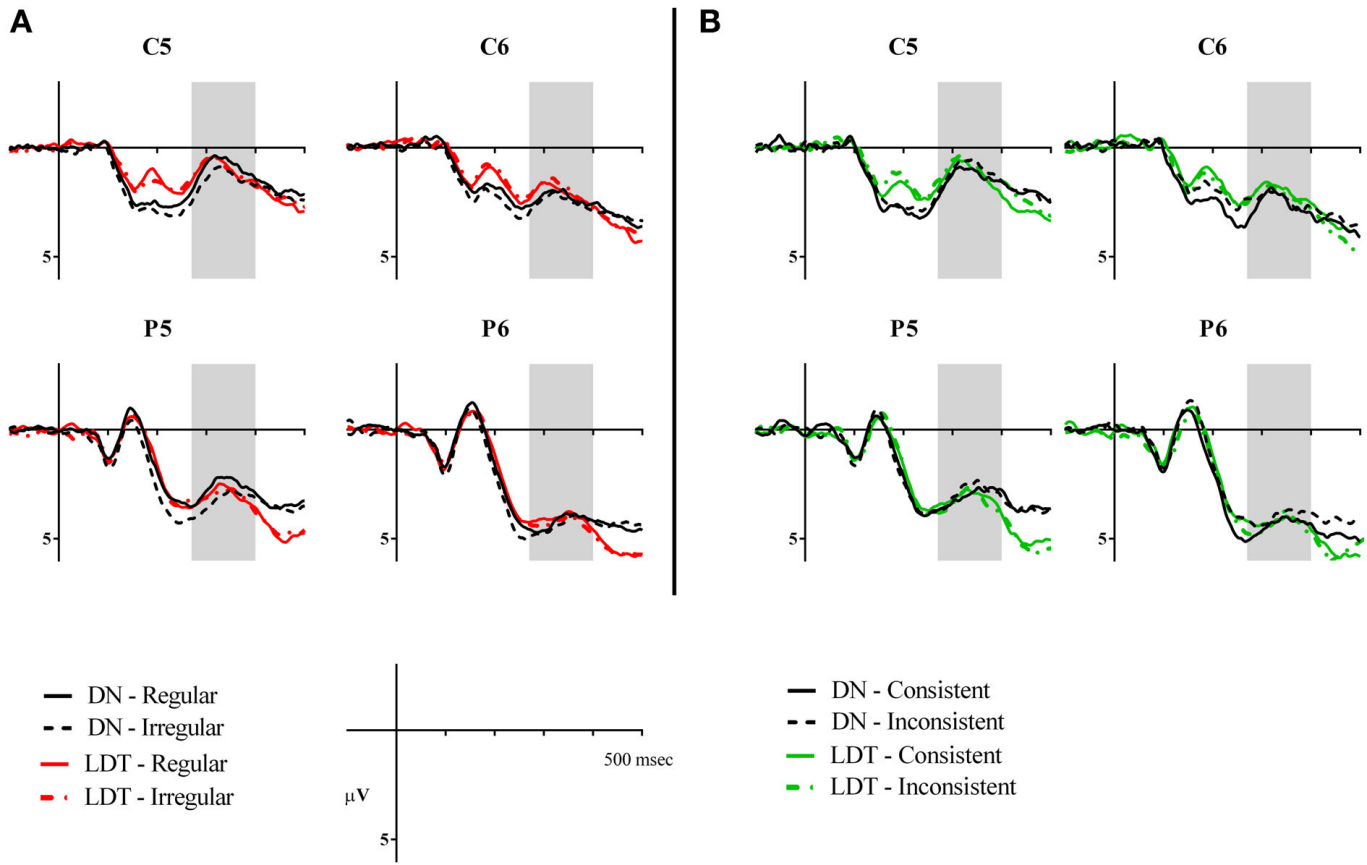
**(A)** Grand average waveforms showing the N400 regularity effect at central and parietal sites (C5, C6, P5, and P6). **(B)** Grand average waveforms showing the N400 consistency effect at the same electrodes. Shaded areas represent the analysis window of 270–400 ms.

**Figure 6:**
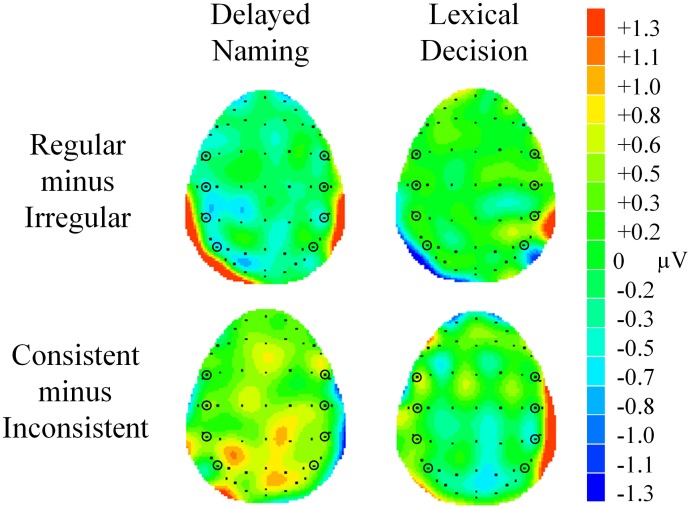
**Voltage maps showing effects of regularity and consistency in delayed naming and lexical decision in N400**. The maps were calculated from the mean difference values (in μV) of regular minus irregular characters and consistent minus inconsistent characters between 270–400 ms. Electrodes FC5, FC6, C5, C6, CP5, CP6, P5, and P6 are circled for reference.

**Figure 7:**
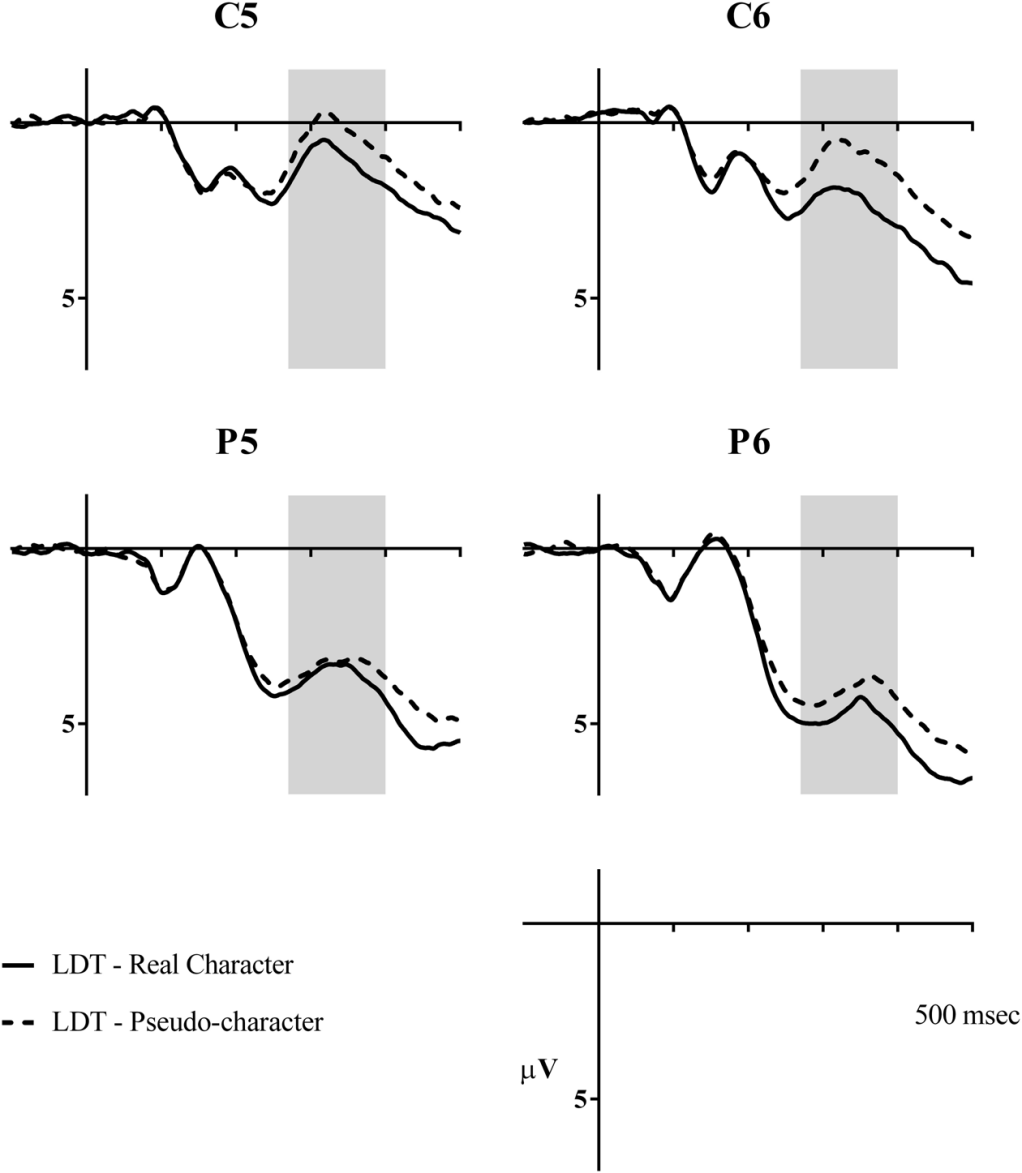
**Grand average waveforms showing the N400 lexicality effect at central and parietal sites (C5, C6, P5, and P6)**. Shaded areas represent the analysis window of 270–400 ms.

**Figure 8:**
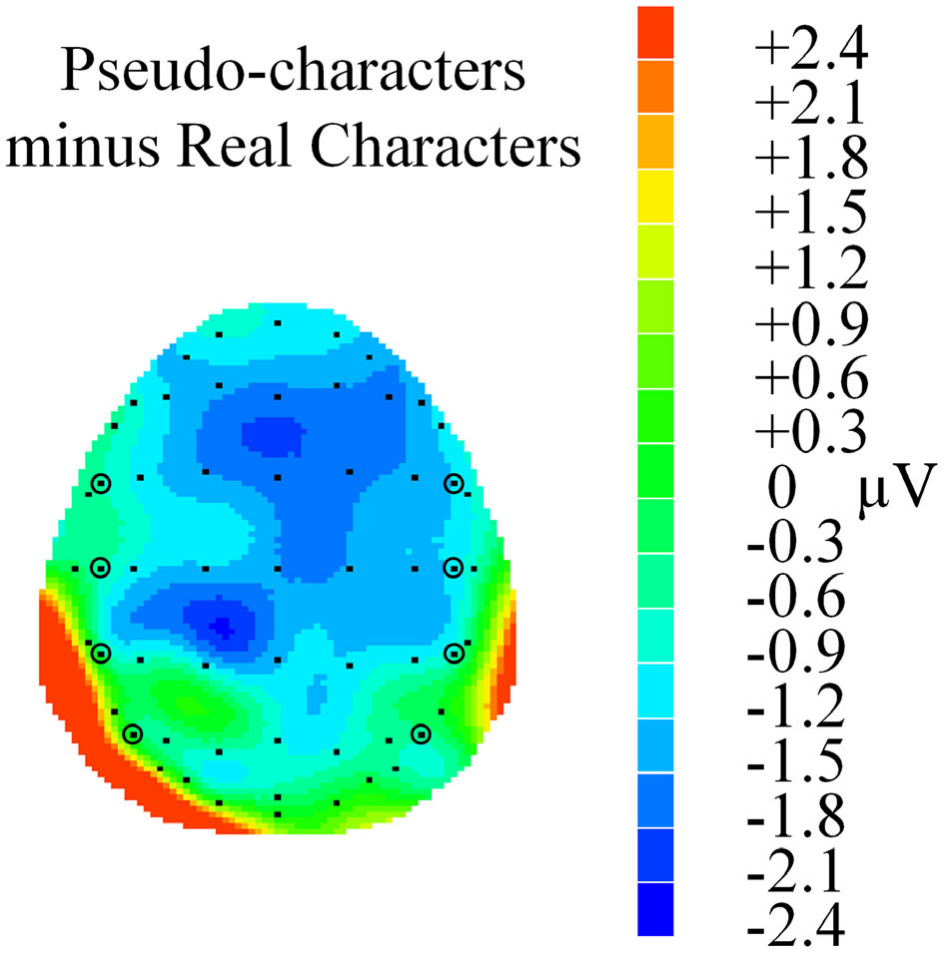
**Voltage map showing the lexicality effect in the lexical decision task in N400**. The map was calculated from the mean difference (in μV) of pseudo-characters minus real characters between 270–400 ms. Electrodes FC5, FC6, C5, C6, CP5, CP6, P5, and P6 are circled for reference.

On average, 10.4% of trials were rejected due to incorrect responses or other artifacts. LD had more remaining trials than DN in both the regularity contrast (*M* = 50.5 vs. 47.8) and the consistency contrast (*M* = 33.5 vs. 31.1). However, the numbers of trials for regular and irregular characters and for consistent and inconsistent characters were comparable in each task. The grand average waveforms and voltage maps for the consistency and regularity contrasts at N170, P200, and N400 time windows are plotted in Figures 1–6. Those for the lexicality contrast at the N400 window are shown in Figures 7, 8, respectively.

#### N170 (100–200 ms)

A significant regularity × task interaction was seen in this component [*F*_(1, 23)_ = 9.24, *p* = 0.006, ηρ^2^ = 0.29). A larger N170 was seen in regular characters than irregular characters in DN only (regular: *M* = −0.68, *SE* = 0.53; irregular: *M* = −0.32, *SE* = 0.60, *p* = 0.014) but not in LD (regular: *M* = −0.47, *SE* = 0.43; irregular: *M* = −0.51, *SE* = 0.51, *p* > 0.05), see Figure 1A and Figure 2. Consistency effects were also observed in a consistency x task x hemisphere interaction [*F*_(1, 23)_ = 6.90, *p* = 0.015, ηρ^2^ = 0.23]. Examination of Figures 1B, 2 suggested consistent characters elicited a larger N170 than inconsistent characters in the right hemisphere in LD. However, follow-up *post-hoc* analyses did not reveal significant two-way interactions or pairwise differences in any of the conditions.

#### P200 (200–270 ms)

A main effect of regularity was found [*F*_(1, 23)_ = 4.64, *p* = 0.042, ηρ^2^ = 0.17], with irregular characters (*M* = 2.90, *SE* = 0.32) eliciting significantly larger P200 than regular characters (*M* = 2.67, *SE* = 0.33). This regularity effect was marginally modulated by task [*F*_(1, 23)_ = 3.56, *p* = 0.072, ηρ^2^ = 0.13]. Irregular characters were more positive than regular characters in DN (regular: *M* = 2.94, *SE* = 0.39; irregular: *M* = 3.50, *SE* = 0.40, *p* = 0.012) but not in LD (regular: *M* = 2.39, *SE* = 0.33; irregular: *M* = 2.30, *SE* = 0.33, *p* > 0.05), see Figures 3A, 4. As for effects of consistency, a consistency x task interaction was seen in this time window [*F*_(1, 23)_ = 4.97, *p* = 0.036, ηρ^2^ = 0.18]. Participants showed larger P200 in response to consistent characters than inconsistent characters in DN (consistent: *M* = 3.56, *SE* = 0.42; inconsistent: *M* = 2.95, *SE* = 0.37, *p* = 0.024) but not in LD (consistent: *M* = 2.65, *SE* = 0.37; inconsistent: *M* = 2.62, *SE* = 0.35, *p* > 0.05), see Figures 3B, 4. Furthermore, a consistency × hemisphere × electrode interaction was found [*F*_(2, 46)_ = 4.87, *p* = 0.016, ηρ^2^ = 0.18]. Although it appeared that the consistency effect was stronger in the right hemisphere, follow-up analyses did not reveal significant differences in two-way interactions or pairwise comparisons.

#### N400 (270–400 ms)

A main effect of regularity was obtained [*F*_(1, 23)_ = 4.53, *p* = 0.044, ηρ^2^ = 0.17]. Significantly larger N400 was elicited by regular characters (*M* = 2.12, *SE* = 0.26) than irregular characters (*M* = 2.32, *SE* = 0.30), see Figures 5A, 6. No other significant effects were obtained. There were also null effects of consistency in this component, see Figures 5B, 6.

A main effect of lexicality was also seen in this window [*F*_(1, 23)_ = 51.90, *p* < 0.001, ηρ^2^ = 0.69]. The N400 for pseudo-characters (*M* = 1.57, *SE* = 0.29) was much larger than for real characters (*M* = 2.31, *SE* = 0.29), see Figures 7, 8. A lexicality by hemisphere interaction was also observed [*F*_(1, 23)_ = 4.84, *p* = 0.038, ηρ^2^ = 0.17]. *Post-hoc* comparisons showed that the lexicality effect was stronger at right hemisphere electrodes (real: *M* = 2.81, *SE* = 0.34; pseudo: *M* = 1.81, *SE* = 0.36, *p* < 0.001), but also significant at left hemisphere electrodes (real: *M* = 1.80, *SE* = 0.34; pseudo: *M* = 1.33, *SE* = 0.33, *p* = 0.012). Furthermore, lexicality interacted with electrode locations [*F*_(3, 69)_ = 10.59, *p* < 0.001, ηρ^2^ = 0.32], but *post-hoc* analyses revealed significant differences at all electrode sites (all *p* < 0.001).

## Discussion

The current investigation examined the independence of regularity and consistency effects during Chinese character recognition using behavioral and ERP measures, and how access to phonological information may be affected by task demands employing LD and DN tasks. It differed from previous reports in that both ortho-phonological effects were studied using ERPs and the patterns of these effects were contrasted between a task explicitly requiring phonological access and one without.

While the main foci of this study were on effects of regularity and consistency and their manifestation as a function of task, the results of lexicality effects in LD would ensure that the participants engaged in the task and the observations of the phonological effects from that task were reliable and valid. Our participants responded to real characters more quickly and accurately than pseudo-characters (546 ms and 97% vs. 661 ms and 89%, respectively). Moreover, they exhibited the typical pattern of greater negativity in N400 to pseudo- than real characters (Bentin et al., 1985; Holcomb, 1993; Nobre and McCarthy, 1994).

Although the behavioral measures concerning regularity and consistency effects were not significant, the ERP results clearly demonstrated the independence of regularity and consistency in terms of different time courses and directions of the effects in specific ERP components, mainly in DN. The effect of regularity, as predicted, was evident very early on in the N170 time window, followed by P200 and N400. Compared with irregular characters, regular characters exhibited more negative N170, less positive P200, and more negative N400. On the contrary, the consistency contrast was reliable only in P200, importantly with consistent characters eliciting more positive response than inconsistent ones. These findings reflect that the two effects have distinct neural correlates. The interaction of these effects with task in N170 and P200, with null effects of regularity and consistency in LD, indicates that phonological information is not automatically accessed during character recognition. Our observations differed most notably from previous work on the consistency effect on character naming in that it was detected only in P200 and was stronger for consistent characters, compared with the presence of the effect in N170, P200, and N400, and more positive P200 for inconsistent characters as reported in Hsu et al. (2009). We have questioned earlier whether consistency in previous ERP studies was confounded with regularity as the characters of the “consistent” condition had a consistency value very close to 1. If the consistency contrast in those studies was indeed one of regularity, i.e., consistent being equivalent to regular and inconsistent to irregular, then the present findings of regularity effects have exactly the same time course and pattern across the three ERP components as “consistency” effects in Hsu et al. (2009) with phonograms of large family size and with neighborhood characteristics well controlled for.

The regularity effect emerged within 200 ms post-stimulus onset. Its occurrence reflects the presence of conflict between phonological forms in irregular phonograms, i.e., those of the phonogram and its phonetic radical. The effect in N170 entails orthographic analysis of the phonogram into its radicals and mapping from the character and stand-alone radical(s) to phonology. This ERP component has been associated with identification of radicals (e.g., Hsiao et al., 2007; Su et al., 2012). Phonological modulations during character recognition on N170 have also been documented, although described as an effect of consistency (Lee et al., 2007; Hsu et al., 2009). More negative N170 for regular characters may reflect greater activation of a single phonological form or facility in processing because of an absence of conflict. The following component, P200, exhibited greater positivity for irregular characters. The direction of the contrast between regular and irregular characters is compatible with that of “consistency” effect in Hsu et al. (2009), as well as regularity effects in English (e.g., Sereno et al., 1998). Stronger P200 may be interpreted as more effortful processing due to competition between two phonological forms. Finally, the observation of more negative N400 for regular than irregular phonograms complements the findings in Lee et al. (2006b) of earlier onset and longer duration of N400 semantic priming effects elicited by regular phonograms. The present result can likewise be interpreted as interaction between phonological and semantic information in this time window. It reflects greater processing effort when different word meanings are mapped onto the same phonological form. One may argue that this account would be relevant to a task that explicitly accesses phonology, i.e., DN, but not necessarily LD. Although the interaction between task and regularity did not reach significance (*p* = 0.101) and only a main effect of regularity was found, inspection of Figures 5, 6 reveals a tendency of greater negativity for regular than irregular characters in DN, particularly for left hemisphere electrodes, but minimal difference in LD.

Reliable effects of consistency were only obtained in P200. Their later appearance, compared with regularity effects, can be explained in terms of competition among orthographic neighbors induced by the phonetic radical of the target phonogram. The identification of the phonetic radical, which takes place during the N170 time window, must precede the activation of phonograms sharing that radical. The shorter duration of the consistency effect may be attributed to the fact that activation of the orthographic neighbors is not sustained by orthographic forms in the stimulus, and the assumption that further access to semantics by activated non-target phonograms is irrelevant to a naming task. The opposite effects of regularity and consistency on P200 have provided critical evidence for their distinction. The result seems counterintuitive in that consistent characters showed greater P200 than inconsistent ones. Note that the contrast between consistent and inconsistent phonograms in this study was a matter of degree. To distinguish consistency from regularity, we included an equal number of regular and irregular characters for the consistent and inconsistent conditions. The consistency values by type or token (Table 1) of phonograms in the “consistent” condition were far from 1, differing from previous investigations. The stimuli in the two consistency conditions were matched in number of stroke, character frequency, orthographic and phonological neighborhood sizes. However, as one would expect, inconsistent characters had more phonological alternatives than consistent phonograms. Following the reasoning of greater competition revealed in stronger P200 in the case of regularity, we propose that fewer phonological competitors actually induce stronger inhibition among one another than when there are more competitors. In the former situation, each phonological form is activated by a larger number of phonograms, while in the latter, activation or competition is more distributed, resulting in weaker mutual interference. Our account is contrary to the one in Lee et al. (2006a) where greater P200 was found for inconsistent characters because more phonological candidates were activated initially. We have tried to argue in this paper that consistency was conflated with regularity in Lee et al. (2006a, 2007) and Hsu et al. (2009), and our findings of regularity parallel theirs of consistency in P200.

The present findings, together with those reported in the works by Lee and colleagues, have clearly demonstrated that skilled readers of Chinese can access phonology from characters within 200 ms in a reading task. This observation differs dramatically from studies of alphabetic scripts, which have traditionally focused on late components. They include the N400 and the following late positive complex (LPC), which occurs between 500 and 800 ms over the left centro-parietal region and is generally interpreted as reflecting conflict resolution and word recognition memory (see Rugg and Curran, 2007; Van Petten and Luka, 2012 for review). The interest in late components might be due to the fact that in the alphabetic writing system whole-word phonology is only available upon or after lexical access, which is believed to take place at the N400 time window (see Lau et al., 2008; Kutas and Federmeier, 2011 for review). However, as argued in Sereno and Rayner (2003), reading research employing the eye movement method has consistently found that the average fixation duration of normal adult readers is around 250 ms. Hence, the focus on N400 and LPC is unlikely to reveal the full picture of online stages of processing during word recognition.

Ample evidence has been accumulated to establish the N170 as an index of one's sensitivity to print (see Maurer and McCandliss, 2007 for review). The reading-related N170 is obtained when words, pseudowords, and letter strings are contrasted with non-linguistic visual forms, and left lateralization of the component is indicative of reading expertise (Maurer et al., 2008). Maurer and McCandliss (2007) have further put forth the phonological mapping hypothesis that the degree of lateralization of N170 may be correlated with the depth of an orthographic system. To illustrate, readers of German, a transparent script, showed comparable left-lateralized N170 to real words and pseudowords, while readers of English, a more opaque script, exhibited stronger effects for words than pseudowords (Maurer et al., 2005). Maurer and colleagues proposed that the left lateralization of N170 is related to the exposure to grapheme-phoneme conversion during reading acquisition. Hence, one would not expect a left-lateralized N170 in readers of a logographic system such as Chinese. The prediction seems to find support from Kim et al. (2004) in which native Korean speakers learning English and Chinese as second languages (L2) were presented with words in these languages as well as pictures in a semantic categorization task. Left-lateralized N170 responses were observed for Korean and English, while bilateral distribution of the component was seen for Chinese and pictures. However, as little information was provided for the participants' proficiency in English and Chinese, it is not clear whether differential responses were due to the properties or the levels of proficiencies of their L2s. In fact, Maurer et al. (2008) found stronger N170 in the left hemisphere of Japanese native speakers to all three Japanese scripts, i.e., *katakana*, *hiragana*, *kanji* characters, compared to their less familiar English script. In studies involving native Chinese readers, the results are mixed with respect to the laterality of N170. Lee et al. (2007), Hsu et al. (2009), as well as the present study, did not find hemispheric dominance of the component; nonetheless, left-lateralization of the N170 has been obtained in adults (Lin et al., 2011; Zhao et al., 2012) and children (Cao et al., 2011; Su et al., 2013). In sum, the N170 has consistently been associated with skilled reading in different writing systems, and left-lateralization of the component is not necessarily influenced by the nature of orthography-phonology mapping.

ERP studies of alphabetic scripts showing effects of GPC on P200 and N400 are equally few. Sereno et al. (1998) obtained greater P200 peaking at 168 ms post-stimulus in the centro-frontal region for low frequency regular than exception English words in a LD task. However, the effect was observed from a subset, 13 out of 32, of the participants. The interaction between phonology and orthography reflected in N400 seems to be restricted to pseudohomophones, i.e., pseudowords that sound like real words. Briesemeister et al. (2009) found weaker effects of pseudohomophones in contrast with pseudoword controls in N400 in LD of German words. Comparable facilitative effects in N400 were shown for pseudohomophones and semantically related words, compared with semantically incongruent words and pseudowords, in the context of semantically constrained sentences (Newman and Connolly, 2004). Although results of these two studies suggested that phonological information generated from print played a role in reading during the N400 time window, they did not come from a direct contrast of regularity or consistency.

The influence of orthography-phonology mapping on word reading in Chinese and the alphabetic writing system is shown to be very different, as evidenced by the manifestations of the effects from early to late ERP components. In Chinese, the regularity effect occurs simultaneously as orthographic analysis begins to take place and persists until lexical recognition; its emergence is immediately followed by the relatively short-lived consistency effect. On the other hand, little evidence points to early occurrence of these effects in alphabetic scripts. This contrast seems counterintuitive at first glance. We propose that the different time courses of access to phonology from print stems from a fundamental difference in the nature of orthography-phonology mapping between the two orthographic systems, namely, addressed phonology in the logographic system and inherently assembled phonology in alphabetic scripts. As described in the Introduction and argued in Law et al. (2009), phonological access is always lexical in Chinese word processing. In the contrast of regularity, the competition is between the pronunciation of the phonogram and that of its phonetic radical as a character. In the case of consistency, the competition is among phonological forms of phonograms sharing the same phonetic radical, regardless of the lexical status of the phonetic radical. Our view differs from the two-stage framework consisting of sublexical and lexical processing indexed by the P200 and N400, respectively (Lee et al., 2007). However, we believe that the divergence is superficial and a matter of wording, since the underlying mechanism of character naming portrayed in Lee et al. (2007) is essentially the same as the one presented here.

In conclusion, the main findings of this investigation, along with those of previous ERP studies of Chinese character reading, have captured a basic difference between logographic and alphabetic writing systems in terms of phonological access from visual word in the early stages of lexical recognition. The conceptual distinction between regularity and consistency in Chinese allowed us to examine their effects independently. The different mechanisms were clarified through their occurrence across ERP components. Finally, the comparison between LD and DN has demonstrated that access to phonological information from print is not automatic and subject to task demands.

## Author contributions

Conceived and designed the experiments: Yen Na Yum, Sam-Po Law, I-Fan Su, and Kai-Yan Dustin Lau. Collected and analyzed the data: Yen Na Yum and Kwan Nok Mo. Interpreted the data: Yen Na Yum, Sam-Po Law, I-Fan Su, Kai-Yan Dustin Lau, and Kwan Nok Mo. Wrote the paper: Yen Na Yum and Sam-Po Law.

### Conflict of interest statement

The authors declare that the research was conducted in the absence of any commercial or financial relationships that could be construed as a potential conflict of interest.
